# Collectanea

**Published:** 1832-07

**Authors:** 


### COLLECTANEA.

**Floriferis ut apes in saltibus omnia libant,**
**Omnia nos, itidem, depascimur aurea dicta.**

### PHYSIOLOGY.

**The following paper, by Dr. Robert Lee, will, we are certain, be perused with much interest. The facts described in it appear to us to refute most completely the opinions of the Hunters respecting the structure of the human placenta, and its connexion with the uterus.**

*On the Structure of the Human Placenta, and its Connexion with the Uterus.* By Robert Lee, M.D. F.R.S. &c., Physician to the British Lying-in Hospital. (From the *Philosophical Transactions.*)

In the year 1780, Mr. John Hunter presented a paper to the Royal Society, in which he laid claim to the discovery of the true structure of the placenta and its communication with the vessels of the uterus. The following is the history of the appearances which he observed in the dissection of a woman who had died undelivered near the full term of utero gestation, and from which appearances his conclusions were drawn respecting the natural structure of these parts. The veins and arteries of the uterus having been injected, an incision was made through the parietes, at the interior part where the placenta adhered to the internal surface. Between the uterus and placenta lay an irregular mass of injected matter, and from this mass regular pieces of the wax passed obliquely between it and the uterus, which broke off, leaving part attached to that mass; and on attentively examining the portions towards the uterus, they plainly appeared to be a continuation of the veins passing from it to this substance, which proved to be the placenta. Other vessels, about the size of a crow-quill, were seen passing in the same manner, although not so obliquely. These also broke on separating the placenta and uterus, leaving a small portion on the surface of the placenta; and, on examination, they were discovered to be continuations of the arteries of the uterus. The veins were next traced into the substance of what appeared placenta; but these soon lost the regularity of vessels, by terminating at once upon the surface of the placenta, in a very fine spongy substance, the interstices of which were filled with yellow injected matter. He then examined the arteries; and tracing them in the same manner towards the placenta, found that, having made a twisted or close spiral turn upon themselves, they were lost on its surface.

On cutting into the placenta, he discovered in many places of its substance yellow injection, and in others red, and in many others these two colours mixed. The substance of the placenta, now filled with injection, had nothing of the vascular appearance nor that of extravasation, but had a regularity in its form which shewed it to be naturally of a cellular structure, fitted to be a reservoir for blood.

From these appearances Mr. Hunter infers, "that the arteries which are not immediately employed in conveying nourishment to the uterus go on towards the placenta, and proceeding obliquely between it and the uterus, pass through the decidua without ramifying. Just before entering the placenta, after making two or three close spiral turns upon themselves, they open at once into its spongy substance, without any diminution of size, and without passing behind the surface as above described.

"The veins of the uterus appropriated to bring back the blood from the placenta, commence from this spongy substance by such wide beginnings as are more than equal to the size of the veins themselves. These veins pass obliquely through the decidua to the uterus, enter its substance obliquely, and immediately communicate with the proper veins of the uterus. This structure of parts points at once to the nature of the blood's motion in the placenta. The blood detached from the common circulation of the mother moves through the placenta of the fœtus, and is then returned back into the course of the circulation of the mother to pass on to the heart."*

Dr. William Hunter's description of the vascular connexion between the uterus and placenta coincides with that of his brother: "for it seems incontestible (he observes) that the human placenta, like that of the quadruped, is composed of two distinct parts, though blended together; viz. an umbilical, which may be considered as a part of the fœtus, and an uterine which belongs to the mother; that each of these parts has its peculiar system of arteries and veins, and its peculiar circulation, receiving blood by its arteries and returning it by its veins; that the circulation through these two parts of the placenta differs in the following manner: in the umbilical portion the arteries terminate in the veins by a continuity of canal, whereas in the uterine portion there are intermediate cells into which the arteries terminate, and from which the veins begin."†

It is a singular fact, that these celebrated anatomists should both have asserted their claims to the merit of what they supposed to be the discovery of the true structure of the human placenta, and its connexion with the uterus, and that their controversy on this subject should have loosened those bonds of affection which had united them together from their earliest years.‡

Noortwych, Rœderer, and Haller, had previously investigated this subject by injecting the blood-vessels of the gravid uterus: their researches however did not determine, in a satisfactory manner, that a vascular connexion exists between the uterus and cells in the placenta. The opinions of the Hunters were generally acquiesced in at the time they were promulgated, and their accuracy has not been called in question by any anatomist of reputation in this country for the last forty years.

In the communication which I have now the honour of presenting to the Royal Society, I propose to describe certain appearances which I have observed in the examination of six gravid uteri,^ and many placentæ expelled in natural

* Observations on certain Parts of the Animal Œconomy, by John Hunter, 1786; page 127.

† Anatomical Description of the Gravid Uterus and its Contents, by the late W. Hunter, M.D. London, 1794; page 48.

‡ Their letters are preserved in the Archives of the Royal Society.

^ in which the natural relation of the placenta to the Uterus had not been destroyed by the forcible injection of extraneous matter into the spermatic & hypogastric arteries. — The accuracy of this description [illegible] by the appearances observed in labour, which seem to demonstrate that a cellular structure does not exist in the placenta, and that there is no connexion between this organ and the uterus by great arteries and veins.

If an incision be made through the parietes of the gravid uterus, where the placenta does not adhere, the membrana decidua will be observed lining the internal surface, and numerous minute blood-vessels and fibres passing from the inner membrane of the uterus to the decidua. At the circumference of the placenta, the membrana decidua separates from the chorion and amnion to pass between the uterus and placenta, and thus forms a complete membranous septum, which is interposed betwixt these organs. The chorion and amnion cover the fœtal surface of the placenta; and between these two membranes and the decidua lie the ramifications of the umbilical vein, and arteries subdivided to an almost indefinite extent, and connected together by white slender filaments running in various directions. The placenta thus consists solely of a congeries of the umbilical vessels, covered on the fœtal surface by the chorion and amnion, and on the uterine surface by the deciduous membrane, and enclosed between these membranes; it adheres to the fundus, or some part of the uterus, by innumerable flocculent fibres and vessels.

On detaching the placenta carefully from the uterus, the deciduous membrane is found to adhere so closely to the umbilical vessels which it covers, that it is impossible to remove it without tearing these vessels. With the fibres uniting the placental decidua to the uterus are mingled numerous small blood-vessels, proceeding from the inner membrane of the uterus to the decidua; and these vessels, though more numerous at the connexion of the placenta with the uterus, exist universally throughout the whole extent of the membrane. There is no vestige of the passage of any great blood-vessel, either artery or vein, through the intervening decidua, from the uterus to the placenta; nor has the appearance of the orifice of a vessel been discovered, even with the help of a magnifier, on the uterine surface of the placenta. This surface of the placenta, deprived of the deciduous membrane, presents a mass of floating vessels, its texture being extremely soft and easily torn; and no cells are discernible in its structure, by the minutest examination.

At that part of the surface of the uterus to which the placenta has been adherent, there are observable a great number of openings leading obliquely through the inner membrane of the uterus, and large enough to admit the point of the little finger: their edges are perfectly smooth, and present not the slightest appearance of having been lacerated by the removal of the placenta. In some places they have a semilunar or elliptical form, and in others they resemble a double valvular aperture. Over these openings in the inner membrane of the uterus, the placenta, covered by deciduous membrane, is directly applied, and closes them in such a manner that the maternal blood, as it flows in the uterine sinuses, cannot possibly escape either into the cavity of the uterus, or into the substance of the placenta. The above appearances on the inner surface of the uterus have been accurately represented by Rœderer; from whose work, fig. 1 of Plate I. is taken.

When air is forcibly thrown either into the spermatic arteries or veins, the whole inner membrane of the uterus is raised by it; but none of the air passes across the deciduous membrane into the placenta, nor does it escape from the semilunar openings in the inner membrane of the uterus, until the attachment of the deciduous membrane to the uterus is destroyed. There are no openings in the deciduous membrane corresponding with these valvular apertures now described, in the internal membrane of the uterus. The uterine surface of the placenta is accurately represented in fig. 2, Plate I.

If a placenta be examined which has recently been separated from the uterus in natural labour, without any artificial force having been employed, its surface will be found uniformly smooth, and covered with the deciduous membrane;

The vascular preparations of the gravid uterus in the Hunterian Collection at Glasgow & in London & by Mr Owen's dissections of the Uterus & pla[illegible]

[illegible]

which could not be the case did any large vessels connect it with the uterus. The placenta in a great majority of cases is also detached from the uterus after labour, with the least imaginable force; which would be impossible if an union by large blood-vessels, possessing the ordinary strength of arteries and veins, actually existed. Besides, a vascular connexion of such a kind would be likely to give rise, in every case, to dangerous hemorrhage subsequent to parturition, a circumstance not in accordance with daily experience.

NOORTWYCH, RŒDERER, HALLER, Dr. W. and Mr. J. HUNTER and Dr. DONALD MONRO, do not appear to have examined the gravid uterus and its contents in the natural state of the parts, but after fluids had been forcibly injected into the hypogastric and spermatic arteries. The laceration of the deciduous membrane covering the orifices of the uterine sinuses followed this artificial process, as well as the formation of deposits of injection in the vascular structure of the placenta, giving rise to the deceptive appearance of cells. That this took place in the examinations made by RŒDERER* and MONRO,† does not admit of dispute; and the following facts render it more than probable that the HUNTERS were also misled, by the effects of artificial distention of the placenta, from the extravasation of the fluids forced into the uterine vessels.

In the course of last autumn, the preparations of the gravid uterus in the Hunterian Museum at Glasgow were examined at my request by Dr. NIMMO; and in none of them does it appear certain that any great blood-vessels pass from the uterus into cells in the placenta; but in many the deposits of injection, causing the appearance of cells, were observed evidently to be the result of extravasation. No preparation in the collection seems to have been expressly made for the purpose of proving or disproving the fact that the deciduous membrane passes over the uterine surface of the placenta; but in reference to preparation R. R. No. 139, it is observed by Dr. NIMMO that no vascular openings are visible in the membrane interposed between the uterus and placenta.

No. 178. "is a small section of the uterus with the veins injected green, and broken off where they were entering the placenta." The surface of the injected matter is smooth; the edges of the openings defined, and quite unlike ruptured vessels; their form in general elliptical, seeming as if they were holes cut in the side of a convolution.

No. 125. "A portion of uterus and placenta, the latter injected from uterine vessels." There is an opening which seems to be natural, corresponding to one of those in the uterus; but the majority of those whereby the injection has passed into the placenta seem to be mere lacerations.

No. 101. "A section of uterus with veins injected black, and the injected matter protruding by irregular plugs into the cavity of the uterus." The holes are semilunar and elliptical, with defined edges, and nothing resembling the continuation of vascular tubes to be seen.

R. R. 121, is described in the printed catalogue as follows: "A small portion of placenta and uterus where the cells of the placenta have been injected from the veins of the uterus. The veins are seen very large, entering the substance of the placenta."

Dr. NIMMO makes the following observations on this specimen: "This preparation seems to be most in point. I would describe it differently. The cellular substance of the placenta has certainly been filled from the uterine vessels. These, however, instead of passing directly into the placenta, are distinctly seen applying their open mouths to the membrane of the placenta, where the injection in some instances stops. The membrane is thinner here than where no vessels are applied, consisting, so to describe it, of one layer, while a second layer covers

* Icones Uteri Humani, Observationibus illustratæ. J. G. Rœderer, 1759.

† Essays and Observations, Physical and Literary, read before a Society in Edinburgh, 1754, vol. i.

all other parts. Where the injection has passed into the substance of the placenta, it has evidently been forced to the side between the layers, and found some weak point, whereby it has entered into and been diffused throughout the cellular texture of the placenta."*

In the Museum of the Royal College of Surgeons of London, there is a preparation of the uterus with the placenta adhering to the inner surface, which is supposed to have been put up by Mr. Hunter himself nearly fifty years ago. The vessels both of the uterus and placenta have been filled with injection, and the parietes of the uterus, placenta, and membranes, have all been divided by a vertical section into two nearly equal portions. By permission of the Board of Curators, I have been enabled to examine one of these portions, and to have a drawing of it made. In the interstices of the muscular fibres I observed the veins of the uterus, which ran in great numbers towards the part where the placenta adhered. They were of an oval form, their long axes being in the long axis of the uterus. The muscular fibres ran longitudinally from the fundus to the os uteri. (Plate II.)

The deciduous membrane was everywhere covered with minute, tortuous blood-vessels proceeding from the inner surface of the uterus, and filled with injection. There was no appearance of vessels of any magnitude passing between the inner surface of the uterus and placenta; but flattened portions of injection were observed in this situation, having in many parts the form of thin layers, which had obviously escaped from the orifices of the uterine veins. Elsewhere the injection had lacerated the deciduous membrane, and formed deposits in the vascular part of the placenta.

The facts which have now been stated warrant, I think, the conclusion, that the human placenta does not consist of two parts, maternal and fœtal, that no cells exist in its substance, and that there is no communication between the uterus and placenta by large arteries and veins. The whole of the blood sent to the uterus by the spermatic and hypogastric arteries, except the small portion supplied to its parietes and to the membrana decidua by the inner membrane of the uterus, flows into the uterine veins or sinuses, and after circulating through them, is returned into the general circulation of the mother by the spermatic and hypogastric veins, without entering the substance of the placenta. The deciduous membrane being interposed between the umbilical vessels and the uterus, whatever changes take place in the fœtal blood, must result from the indirect exposure of this fluid, as it circulates through the placenta, to the maternal blood flowing in the great uterine sinuses.

Since the preceding paper was forwarded to the secretary of the Royal Society, the following valuable communication has been received by the author from Mr. Owen, to whom portions of the gravid uterus and placenta were submitted for minute examination.

*Lincoln's Inn Fields; 17th November.*

My dear Sir: During the time you were examining the Hunterian preparation of the uterus and placenta in the Museum of the Royal College of Surgeons, your observations on the obscurity produced by the extravasated injection led me to think of some less objectionable mode of demonstrating the vascular communication between the uterus and placenta, if it existed; or of proving, more satisfactorily than the appearances you pointed out in that preparation seemed to do, that there was no such communication.

You have since afforded me the means, through the kindness of Mr. Alexander Shaw, of examining, in the manner I wished, the anatomical relations between

* My friend Samuel Broughton, Esq. F.R.S., during a recent visit to the Hunterian Museum at Glasgow, examined the preparations of the placenta and uterus at my request, and authorises me to say that his observations fully confirm the accuracy of Dr. Nimmo's statements.

the placenta and uterus. This has been done by dissecting the parts under water before disturbing them, either by throwing forcibly foreign matter into the vessels, or by separating the placenta from the uterus to observe the appearances presented by the opposed surfaces, a proceeding which, if done in the air, is liable to the objection of the possibility of having torn the vessels which were passing across, and the coats of which are acknowledged, by those who maintain the existence of such vessels, to be extremely delicate.

The mode, therefore, which was adopted to avoid these objections, was to fix under water, in an apparatus used for dissecting mollusca, &c., a section of the uterus and placenta, and, commencing the dissection from the outside, to remove successively and with care, the layers of fibres, and trace the veins as they pass deeper and deeper in the substance of the uterus in their course to the deciduous membrane; in which situation as the thinnest pellicle of membrane is rendered distinct by being supported in the ambient fluid, I naturally hoped in this way to see the coats of the veins continued into the deciduous membrane and placenta, and to be able to preserve the appearance in a preparation, if it actually existed in nature. But in every instance the vein, having reached the inner surface of the uterus, terminated in an open mouth on that aspect; the peripheral portion of the coat of the vein, or that next the uterus, ending in a well-defined and smooth semicircular margin, the central part adhering to, and being apparently continuous with, the decidua.

In the course of this dissection I observed that where the veins of different planes communicated with each other, the central portion of the parietes of the superficial vein invariably projected in a semilunar form into the deeper-seated one; and where (as was frequently the case, and especially at the point of termination on the inner surface,) two or even three veins communicated with a deeper-seated one at the same point, these semilunar edges decussated each other so as to allow only a very small part of the deep-seated vein to be seen. I need not observe to you how admirably this structure is adapted to ensure the effect of arresting the current of blood through these passages, upon the contraction of the fibres with which they are everywhere surrounded.

On another portion of the same uterus and placenta, (which was removed from a woman who died at about the fifth month of utero-gestation,) I commenced the examination under water by turning the placenta and deciduous membrane from the inner surface of the uterus. In this way the small tortuous arteries that enter the deciduous membrane were readily distinguishable, though not filled with injected matter; and as it was an object to avoid unnecessary force in the process of separation, they were cut through, though they are easily torn from the decidua. But with respect to the veins, they invariably presented the same appearances as were noticed in the first dissection, terminating in open semicircular orifices, which are closed by the apposition of the deciduous membrane and placenta. This membrane is, however, certainly thinner opposite these orifices than elsewhere; and in some places appeared to be wanting, or adhering to the vein was torn up with it; but in these cases the minute vessels of the placenta only appeared, and never any indication of a vascular trunk or cell commensurate with the size of the vein whose terminal aperture had been lifted up from the part.

The preparation which accompanies this letter shews the termination of a vein on the inner surface of the uterus, and an artery of the decidua cut through, with the corresponding appearances on the surface of the placenta, also the valvular mode in which the veins communicate together in the substance of the uterus.

I remain yours, very truly,

RICHARD OWEN.

